# Pemphigoid gestationis treated with dupilumab

**DOI:** 10.1016/j.jdcr.2023.08.013

**Published:** 2023-09-07

**Authors:** Rita E. Chen, Christine C. Yokoyama, Milan J. Anadkat

**Affiliations:** Division of Dermatology, Department of Medicine, Washington University School of Medicine, St. Louis, Missouri

**Keywords:** corticosteroid-sparing therapy, dupilumab, immunobullous disease, pemphigoid gestationis, pregnancy related rash, pruritus

## Introduction

Pemphigoid gestationis (PG) is a rare blistering disorder (estimated at 1 case per 2000-60,000 pregnancies) that usually develops during the second and third trimesters of pregnancy, but can present at any stage, including postpartum.^1^ Subsequent pregnancies may result in earlier onset of disease manifestation. PG presents with intense pruritus, erythema multiforme-like or urticarial erythematous plaques, and tense bullae emerging in the periumbilical region that can spread to the extremities. Diagnosis is confirmed with direct immunofluorescence staining on skin biopsy, revealing linear deposition of C3 and sometimes IgG along the basement membrane. Antibody titers by enzyme-linked immunosorbent assay against BP180 protein are both sensitive and specific for PG and can be useful in monitoring disease activity.^2^ Pathogenesis involves autoreactive IgG antibodies against BP180 (collagen XVII), which is expressed in hemidesmosomes at the dermoepidermal junction, leading to vesicle or bullae formation.^2^ It is hypothesized that aberrant major histocompatibility class II presentation of BP180 expressed in placental trophoblastic and amniochorionic stromal cells^3^ generates maternal autoantibodies that cross-react with BP180 in the maternal skin, resulting in the characteristic dermatologic findings.

Traditional treatment of PG includes topical and oral corticosteroids, antihistamines, and intravenous immunoglobulins.^4^ Dupilumab is a monoclonal antibody that inhibits the receptor for interleukin 4 and interleukin 13, leading to decreased type 2 inflammation.^5^^,^^6^ Here, we describe a case of recalcitrant PG that was successfully treated with dupilumab.

## Case report

**Fig 1:**
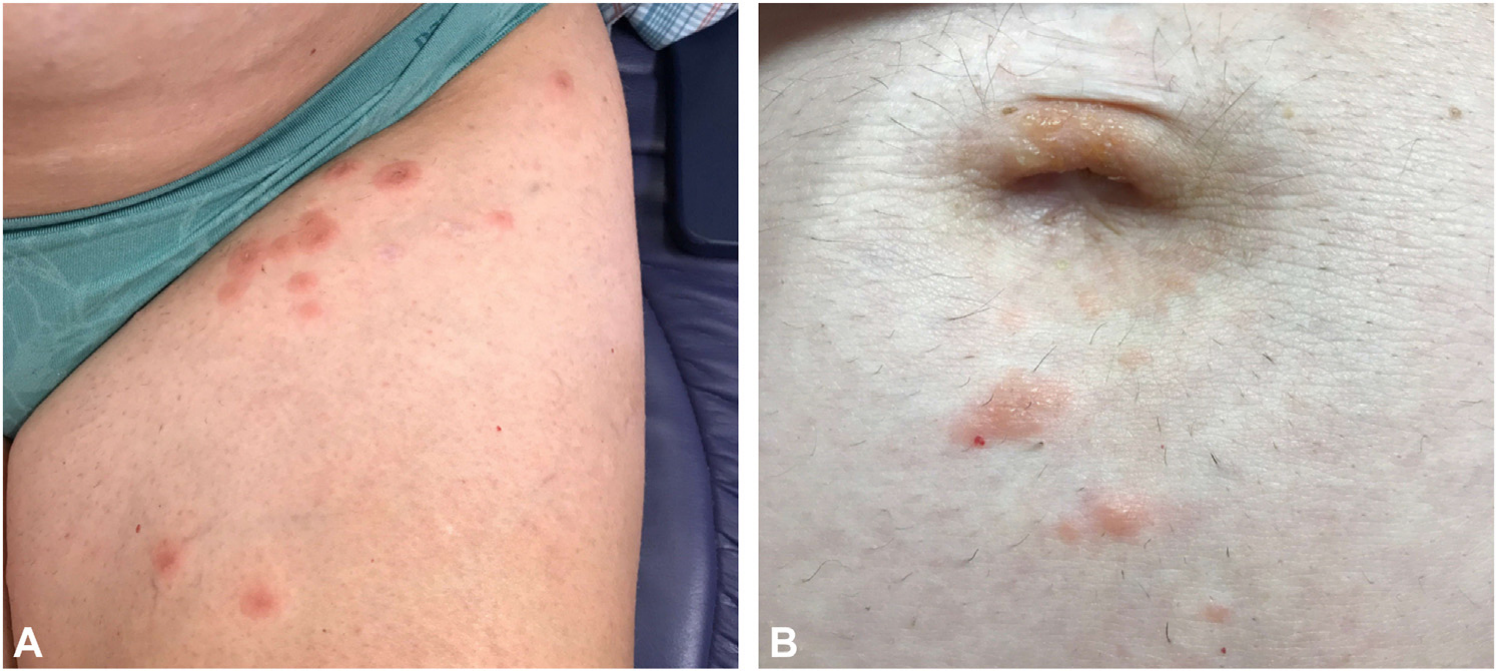
Pemphigoid gestationis rash on presentation. Urticarial plaques on the thigh (**A**). Vesicular lesions near the umbilicus (**B**).

**Fig 2:**
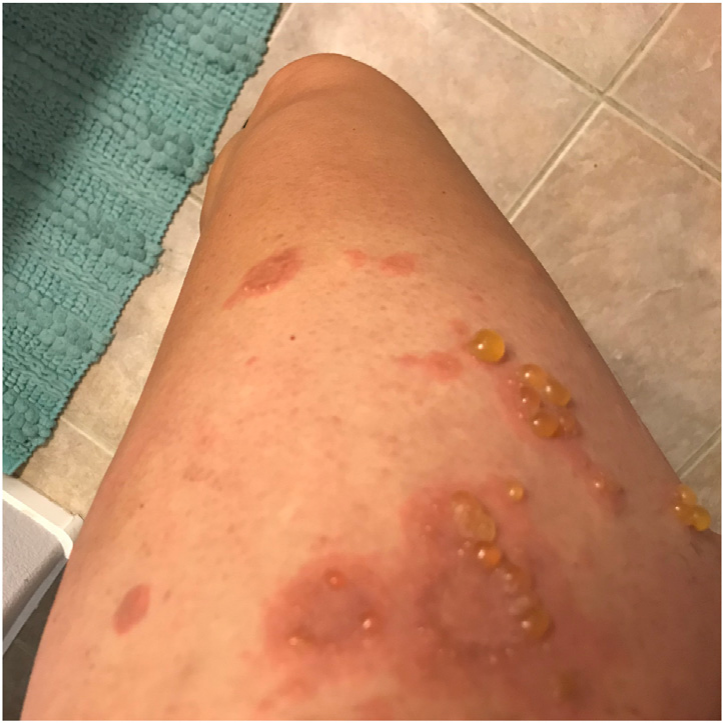
Pemphigoid gestationis rash during first pregnancy. Urticarial plaques and vesicles on the thigh.

**Fig 3:**
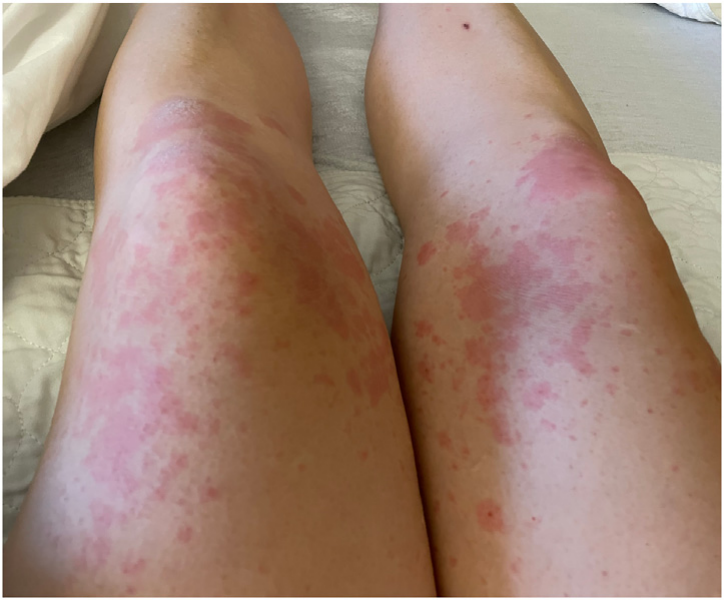
Pemphigoid gestationis rash after delivery. Urticarial plaques on the thighs.

A 36-year-old woman with a prior history of PG was referred to our Dermatology clinic by her obstetrician for an itchy rash on her legs that had been present for 1 to 2 weeks (Fig 1). Her initial episode of PG (Fig 2) manifested at 30 weeks’ gestation in her previous pregnancy 3 years earlier while she was living in a different state; the diagnosis of PG was confirmed by skin biopsy for hematoxylin and eosin and direct immunofluorescence. During the prior pregnancy, her rash was treated with prednisone 40 mg daily until an uneventful induction of labor and delivery of a healthy baby at 39 weeks. She developed pruritus and rash at week 18 of her current pregnancy. Before presentation in our clinic, she had used topical diphenhydramine, topical steroids (hydrocortisone, triamcinolone, and clobetasol), and oral cetirizine without improvement. Skin biopsy was not repeated given her history and detailed records. We instead confirmed diagnosis of PG with an elevated BP180 antibody titer of 90 RU/mL (reference <20 RU/mL). After trial of oral prednisone 40 mg (0.5 mg/kg) daily for 2 weeks, she continued to develop new pruritic blisters. After discussing treatment options, she preferred to avoid increasing oral steroids and wished to pursue steroid-sparing alternatives. We elected to initiate dupilumab with a loading dose of 600 mg administered at 22 weeks’ gestation, followed by 300 mg every 2 weeks. After 4 weeks of dupilumab, she had considerable improvement of rash, blisters, and pruritus. She discontinued oral steroids by week 32. After delivery of a healthy boy at 39 weeks, her rash (Fig 3) continued to resolve with dupilumab alone after 4 weeks postpartum.

## Discussion

PG is a rare pruritic blistering disease that occurs surrounding pregnancy. Treatment primarily relies on oral corticosteroids, which can elicit many undesirable systemic symptoms.^4^ Recently, dupilumab has been shown to be effective as a corticosteroid-sparing treatment for atopic dermatitis, leading to US Food and Drug Administration-approval, and bullous pemphigoid, leading to ongoing clinical trial: reference clinicaltrial.gov NCT04206553.^5^^,^^6^ We hypothesized that dupilumab would succeed in treating our patient’s PG because the pathogenesis of bullous pemphigoid and PG are similar, with each engaging autoantibodies against BP180. There also exist multiple reports of dupilumab use in pregnancy without complication.^7^, ^8^, ^9^ Among the few reported cases, there was no increased risk of major birth defects, miscarriage, or maternal or fetal outcomes in mothers using dupilumab.^7^, ^8^, ^9^ Future studies are warranted to further comment on dupilumab safety during pregnancy. In addition, our case corroborates the findings of a prior report that showed that dupilumab was clinically successful against recurrent PG and also decreased BP180 antibodies.^10^ These case reports illustrate the need to further evaluate dupilumab for PG given the limited treatment options that exist in this setting.

## Conflicts of interest

Dr Anadkat has served as a Consultant and/or Advisor for ImClone, Bristol Myers Squibb, Astra Zeneca, Therakos, Aspire Bariatrics, Biogen, Amgen, Veloce, Adgero, Eli Lilly, Abbvie, UCB Biopharma, Innovaderm, Boehringer-Ingelheim, OnQuality, Novocure, Springworks, BioLinq, and Protagonist. MJA has served as a Principal Investigator for Novartis, Boehringer-Ingelheim, Lutris, OnQuality, UCB Biopharma, InflamRx, Eli Lilly, InCyte, Abbvie, Moonlake, AnaptysBio, Hana Biosciences, Xoma, Veloce, Biogen, Xbiotech, and Chemocentryx. Author Chen and Dr Yokoyama have no conflicts of interest to declare.
